# Bibliometric analysis of the global research status and trends of the association between Vitamin D and infections from 2001 to 2021

**DOI:** 10.3389/fpubh.2022.934106

**Published:** 2022-08-04

**Authors:** Wenfang He, Yali Deng, Xuemei Luo

**Affiliations:** ^1^Department of Critical Care Medicine, The Second Xiangya Hospital, Central South University, Changsha, China; ^2^Department of Obstetrics, The Second Xiangya Hospital, Central South University, Changsha, China; ^3^Department of Pediatrics, The Second Xiangya Hospital, Central South University, Changsha, China

**Keywords:** vitamin D, infectious disease, hot spots, trends, visualization analysis

## Abstract

**Objective:**

The objective of this study was the visualization of hot spots and evolving trends in research on the association between vitamin D and infections through the use of bibliometric analysis.

**Methods:**

Based on 3046 relevant articles collected in the Web of Science Core Collection for the period of 2001–2021, the data were processed using CiteSpace software. GraphPad software was used for some of the graphics.

**Results:**

A total of 3,046 literature were retrieved, with an average citation frequency of 27.89 times. The number of published papers in the direction of “Immunology” (453 articles, 14.9%) and “Infectious diseases” (312 articles, 10.2%) is much higher. The United States presents the highest publication count (890, 29.2%) and shows a strong leadership in this field. Country burst shows that since 2015, many developing countries and low-income countries have carried out enthusiastic research in this regard, including China, Pakistan, and Iran. As for institutions, the League of European Research Universities produces a larger proportion of articles (220, 7.2%). In terms of authors, Martineau AR and Camargo CA have the highest number of published articles, contributing 30 (0.99%) and 28 articles (0.92%), respectively. Major studies are supported by the United States Department of Health Human Services funding (394, 12.9%). According to the keyword co-occurrence diagram, the 10 most frequent keywords from 2001 to 2021 are “vitamin D”, “infection”, “d deficiency”, “risk”, “association”, “expression”, “disease”, “d supplementation”, “vitamin d deficiency”, and “children”. The top 10 cited articles in 2021 are all related to COVID-19, suggesting it is a hotspot in recent times.

**Conclusion:**

Research on the association between vitamin D and infection has grown rapidly since 2012 and is generally developing well. While developed Western countries continue to be leading roles in this field, research trends in developing countries are also very promising. It is demonstrated that the relationship between vitamin D and respiratory infections, especially respiratory viruses and the more recently COVID-19, has received a lot of attention in the last two decades, suggesting that this is the hotspot and frontier of research issue.

## Introduction

As a fat-soluble secosteroid, the two main forms of vitamin D in nature are vitamin D_2_ (ergocalciferol) and vitamin D_3_ (cholecalciferol). Vitamin D_2_ is mainly of plant origin, while vitamin D_3_ accounts for about 80%−90% of the total in higher animals (1). Vitamin D_3_ could be synthesized through exposure to ultraviolet B (UV B) radiation from 7-dehydrocholesterol in the skin (1), which is the major source for most people. If the endogenous synthesis is deficient, usually due to limited skin exposure to sunlight, then dietary supply becomes critical. Both vitamin D_2_ and vitamin D_3_ are inactive and need two consecutive hydroxylation steps to develop fully active vitamin D. Vitamin D is first transported to the liver via vitamin D binding protein (DBP). In the liver, vitamin D_2_ and vitamin D_3_ undergo hydroxylation to 25(OH)D, which is then re-hydroxylated in the kidneys to 1,25(OH)_2_D (calcitriol) (2).

25(OH)D is the main circulating metabolite of vitamin D and the most recognized indicator of vitamin D status currently due to its longer half-life (about 2–3 weeks) (3). Circulating 25(OH)D is tightly bound to DBP (85–90%) or albumin (10–15%), and only a very small fraction is present in free form in the circulation (4). The free hormone hypothesis states that only unbound hormone can be biologically active (5). This hypothesis is supported by observations in DBP-deficient mice. These DBP null mice, although with largely undetectable 25(OH)D levels, did not show signs of vitamin D deficiency unless given a vitamin D-deficient diet (6). It is suggested that DBP is a key reservoir of vitamin D metabolites and may reduce the risk of vitamin D deficiency when ingestion or epidermal production is restricted. Polymorphisms in DBP are associated with disease susceptibility (7).

Similar to other steroid hormones, the active form of vitamin D, 1,25(OH)_2_D, functions by binding to the vitamin D receptor (VDR) to a specific DNA sequence, thereby transcriptionally regulating gene expression and mediating cellular responses (8). VDRs are present in a wide range of cells along with organs, such as the brain, heart, small intestine, colon, osteoblasts, activated T and B lymphocytes, and monocytes (1). Studies also showed that vitamin D can directly or indirectly interact with a wide range of genes (9). The latest study suggested that a dose-dependent alteration in the expression of genes was observed after 25(OH)D supplementation, with 162, 320, and 1,289 genes up- or downregulated, respectively (10). The effects of vitamin D involve anti-proliferation, pro-differentiation, anti-angiogenesis, inhibition of metastasis, and induction of apoptosis in cancer cells (1). Other effects include the increase in insulin secretion, modulation of renin–angiotensin–aldosterone effect, and various immunomodulatory effects, including control of immune activation on the one hand and enhancement of anti-infection defense on the other hand (11–13).

These suggest that vitamin D may play a broad role in human health besides bone health, especially in cancer, cardiovascular disease, diabetes mellitus, and autoimmune diseases (14–16). The latest observational analysis published in *Lancet Diabetes & Endocrinol* suggests a non-linear dose–response relationship between 25(OH)D concentration and cardiovascular disease, stroke, and mortality outcomes (17). Meanwhile, the further genetic analysis for individuals with low concentrations of 25(OH)D provides strong proof supporting a causal relationship between 25(OH)D concentrations and the risk of all-cause mortality at a threshold of approximately 40 nmol/L (17), which is consistent with the previous Mendelian randomization analysis (18). Recent studies also highlight that vitamin D plays an important role in infectious diseases (19, 20). The overall effect of vitamin D deficiency in infections is associated with alteration of the critical immune response such as genetic expression related to antioxidants, cytokine storm, metabolism, and cellular function (21). However, there are arguments that vitamin D does not do much for infections. The coronavirus disease 2019 (COVID-19) epidemic raging around the world has pushed the debate to its climax. Of note, in stark contrast to the importance of vitamin D in health, vitamin D deficiency is prevalent worldwide, regardless of age, ethnicity, latitude, and economic development (22–25). Since the issue has been recognized and taken seriously, the volume of research literature in this direction grows rapidly in recent years. Bibliometric techniques can be used to explore the dynamics of a specialty, mapping from a research frontier to its knowledge base in a time-varying manner (26). With the use of this technique, our studies analyze the relevant data and try to present a realistic and intuitive picture of the evolving trends of research hotspots on the association between vitamin D and infections, to assist a better understanding of the research dynamics in this area.

## Materials and Methods

### Data collection

**Figure 1:**
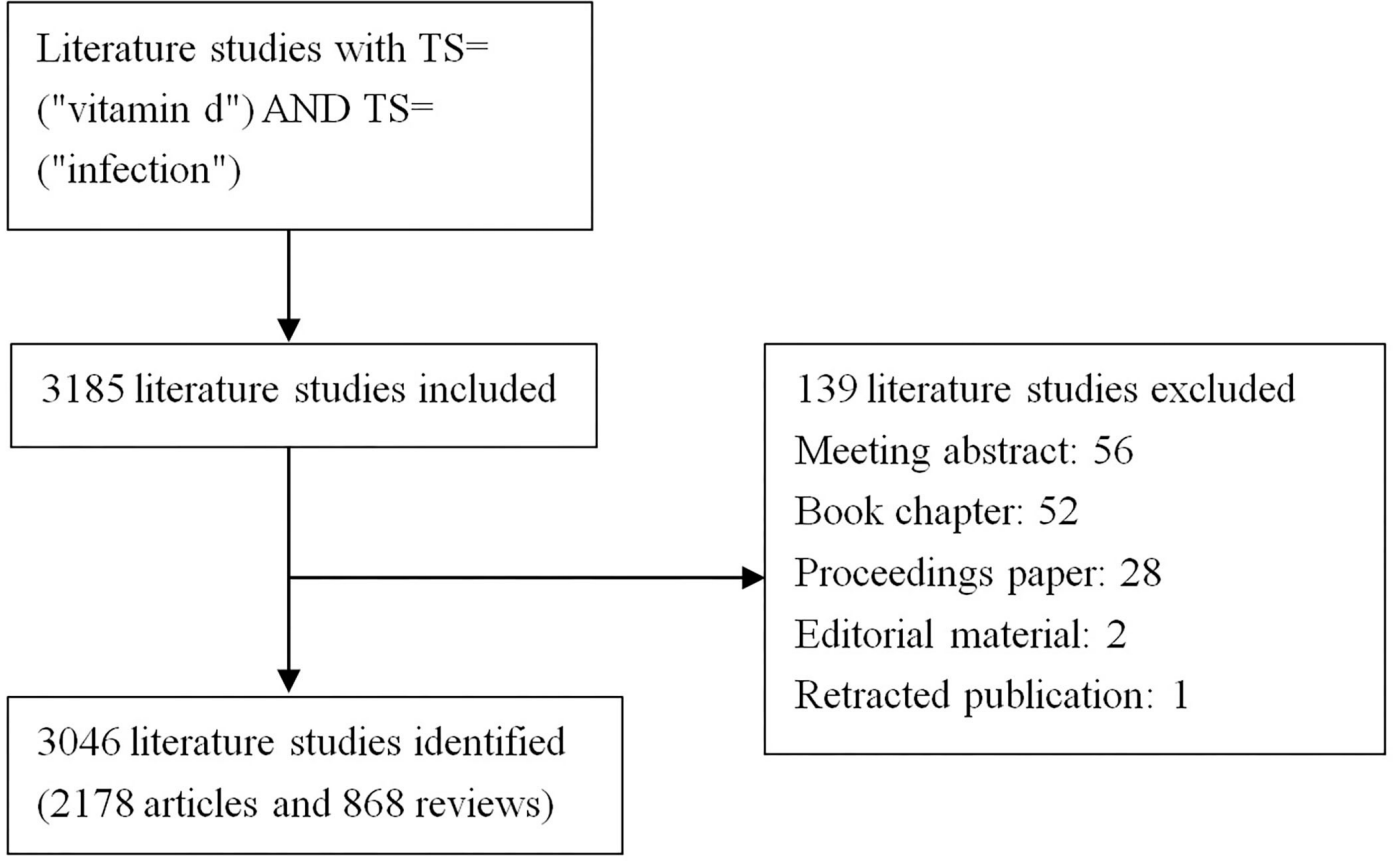
Flowchart of literature screening. Data collection was done on a single day 17 April 2022. A total of 3,185 records from the Web of Science Core Collection (WoSCC) database were retrieved. Then, 139 data were excluded including meeting abstracts, book chapters, proceeding papers, editorial materials, and retracted publications. Finally, 3,046 pieces of data were obtained, including 2,178 articles and 868 reviews. Data were imported into the CiteSpace software (version 5.8.R3) for further analysis.

We performed a systematic search of the literature within the Web of Science Core Collection (WoSCC) database using the strategy described below: TS = (“vitamin d”) AND TS = (“infection”) AND Articles OR Review Articles (Document Types) AND Language = English, with a period limited from 2001 to 2021. To avoid the impact of frequent database updates, all literature searching and data collecting were conducted within 1 day on 17 April 2022. A total of 3,185 records were accessed. Then, we excluded 139 data about meeting abstract, book chapter, proceedings paper, editorial material, and retracted publication. The final search yielded 3,046 papers, including 2,178 articles and 868 reviews. The procedure of searching was presented in Figure 1.

### Data analysis

Retrieved literature data were exported in TXT format and then imported to CiteSpace software (version 5.8.R3) for further analysis and processing. The specific parameters in CiteSpace were set as follows: method (LLR), time slicing (January 2001–December 2020), years per slice (1), term source (title, abstract, author keywords, and keyword plus), node type (select one of the following options at a time: keyword, country), and selection criteria: Top *N* = 50.

The number of publications, major research institutions, leading countries and authors, keywords, and other indicators in the research field of the association between vitamin D and infections was analyzed. By adjusting the relevant parameters, co-occurrence analysis, cluster analysis, and visualization graphs were performed for keywords. In the generated map, centrality was used to reflect the importance of the node in the network. Centrality value > 0.1 was generally considered a comparatively important node. The higher frequency of co-occurrence and higher centrality indicated that the node was more important in this field.

The results of keyword co-occurrence and keyword cluster represented the evolution of research themes in the field over a defined time interval. The result of keyword burst indicated a sharp increase in the intensity of a research direction over different periods, which was used to identify research hotspots. Highly cited articles were summarized and served the same purpose. Country burst showed rapid growth in the number of citations to literature published by that country over this time frame, which was used to indicate the research fervor in a country.

Microsoft Excel (version 2016) and GraphPad software (version 9.3.1) were also applied in data drawing.

## Results

### The global growth trend of publication outputs

**Figure 2:**
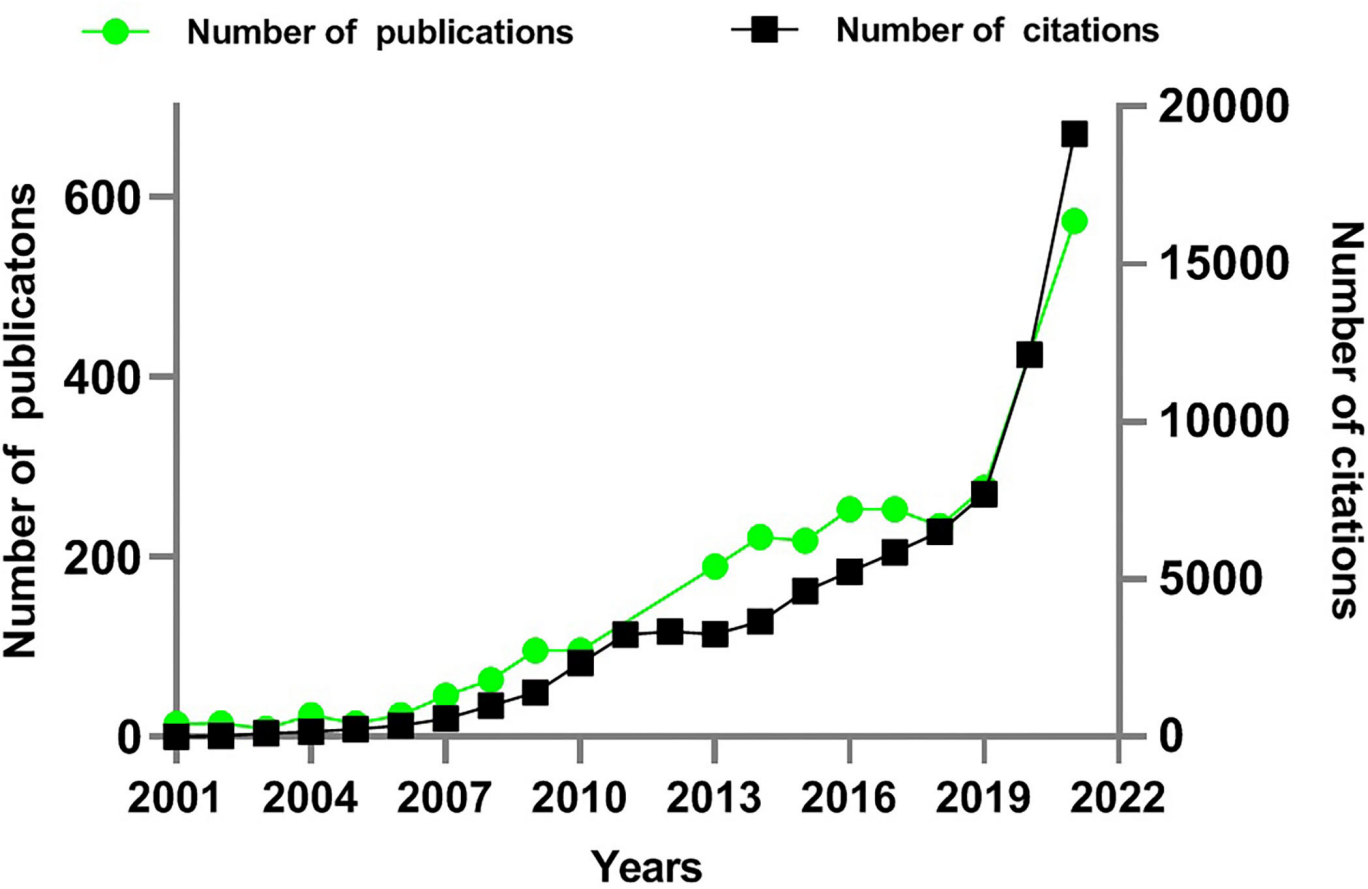
Times cited and publications over time (2001–2021). The green line represents the number of published articles. The black line represents the number of cited articles.

The number of publications is an important index to visualize the trend of the research field. As presented in Figure 2, from 2001 to 2012, there were <100 relevant articles per year on vitamin D and infection. From 2012 onwards, the field has welcomed a rapid growth in the number of literature, reaching 573 articles by 2021. Of note, more than twice as many articles were published in 2021 than in 2019, most possibly due to the sudden outbreak of COVID-19 epidemic. The mean citation frequency is 27.89 times each, and the H-index is 128 times. The cited literature has increased from 3,335 in 2012 to 19,140 in 2021. All these papers cover 82 research directions, with more articles published in the field of “Immunology” (453 articles, 14.9%) and “Infectious Diseases” (312 articles, 10.2%). Other popular areas of research include general internal medicine, nutrition dietetics, and endocrinology metabolism (Table 1).

**Table 1:** Top five based on the number of documents (2001–2021).

**Field**		**Record count**	**% of 3,046**
Research Areas	Immunology	453	14.9
	Infectious Diseases	312	10.2
	General Internal Medicine	311	10.2
	Nutrition Dietetics	274	9.0
	Endocrinology Metabolism	227	7.5
Countries	USA	890	29.2
	England	316	10.4
	Italy	242	7.9
	China	229	7.5
	India	163	5.4
Affiliations	League of European Research Universities	220	7.2
	University of London	133	4.4
	Harvard University	120	3.9
	University of California System	101	3.3
	Egyptian Knowledge Bank	78	2.6
Authors	Martineau AR	30	0.99
	Camargo CA	28	0.92
	Hewison M	18	0.59
	Sun J	17	0.56
	Griffiths CJ	15	0.49
Funding Agencies	United States Department of Health Human Services	394	12.9
	National Institutes of Health	393	12.9
	European Commission	161	5.3
	NIH National Institute of Allergy Infectious Diseases	141	4.6
	UK Research Innovation	96	3.2

### Analysis of country contribution and country burst

**Figure 3:**
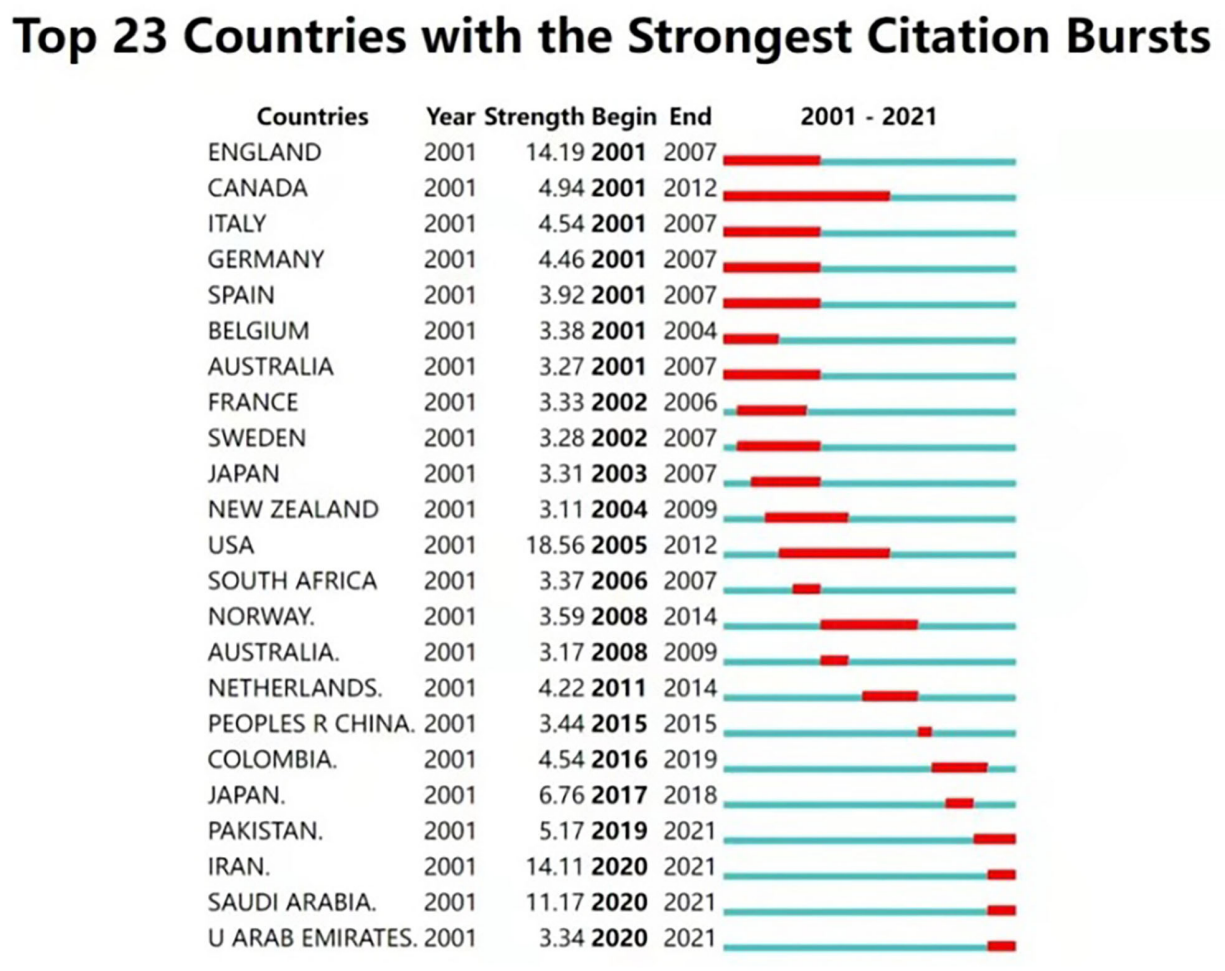
Top 23 countries with the strongest citation bursts (2001–2021). The blue line represents the period, and the red line represents the duration of the citation burst.

A total of 122 countries or regions have contributed to the research on the association between vitamin D and infections. The United States (US) ranks first and leads the way in the number of publications (890, 29.2%), followed by England (316, 10.4%), Italy (242, 7.9%), China (229, 7.5%), and India (163, 5.4%). Table 1 also shows the top five prominent sources of funding. The major funding agencies include the United States Department of Health Human Services and the National Institutes of Health (NIH), all of which are US organizations with approximately the same number of grants. Meanwhile, we further analyzed the strongest citation bursts of publications by country/region from 2001 to 2021 (Figure 3). The result shows that until 2011, the dominant nations were Western developed countries. Since 2015, many developing countries and low-income countries have carried out enthusiastic research in this regard, including China, Pakistan, and Iran. Recently, several Middle Eastern countries have also shown a high enthusiasm for research, such as Saudi Arabia and the United Arab Emirates.

### Analysis of institutions and authors

As shown in Table 1, among the 3,918 institutions contributing to the study of this area, the League of European Research Universities (LERU) published the largest number of papers (220, 7.2%). The following institutions include the University of London (133, 4.4%), Harvard University (120, 3.9%), University of California System (101, 3.3%), and Egyptian Knowledge Bank (78, 2.6%). As for authors, Martineau AR and Camargo CA are the two with the highest number of published articles, contributing 30 (0.99%) and 28 articles (0.92%), respectively. Other authors include Hewison M, Sun J, and Griffiths CJ (Table 1). Their research topics encompass the pathophysiology of vitamin D-related diseases and clinical studies. Interestingly, all these five authors are from universities either in the US or the United Kingdom (United Kingdom). Martineau AR and Griffiths CJ are both colleagues serving at the Queen Mary University of London. Hewison M is at another university in the United Kingdom, namely the Institute of Metabolism and Systems Research, University of Birmingham. Camargo CA works at Massachusetts General Hospital, Harvard Medical School, while Sun J is at the University of Illinois at Chicago.

### Analysis of research topic and frontiers

#### Top ten highly cited articles

Highly cited articles refer to publications with a high citation frequency and a high impact, which could reflect hotspots and depth of research in this field. Table 2 shows the most cited 10 articles in terms of the association between vitamin D and infections. The article published in *Science* by Liu and colleagues in 2006 was the most cited article, with an impressive frequency of 2,599 citations. This article, along with the tenth-ranked literature, discussed the specific mechanisms of vitamin D in the treatment of tuberculosis. Their findings highlighted the critical role of vitamin D in the antimicrobial response in innate immunity. The fifth- and sixth-ranked articles showed the correlation between vitamin D and virus infections and upper respiratory tract infections, respectively. The second-, seventh-, and eighth-ranked articles summarized evidence from randomized controlled studies to investigate whether vitamin D supplementation could prevent viral infections. These highly cited articles illustrate the continued interest in the association of vitamin D with infections over the past two decades.

**Table 2:** Top 10 high-cited references related to vitamin D and infections.

**Ranking**	**Title**	**References**	**Journal**	**Year**	**Cited by**
1	Toll-like receptor triggering of a vitamin D-mediated human antimicrobial response	Liu, PT, et al.	Science	2006	2,599
2	Vitamin D supplementation to prevent acute respiratory tract infections: systematic review and meta-analysis of individual participant data	Martineau, AR, et al.	BMJ-British medical journal	2017	833
3	Genetic dissection of immunity to mycobacteria: The human model	Casanova, JL; Abel, L	Annual review of immunology	2002	736
4	Environmental risk factors for multiple sclerosis. Part I: The role of infection	Ascherio, A; Munger, KL.	Annals of neurology	2004	694
5	Epidemic influenza and vitamin D	Cannell, JJ, et al.	Epidemiology and infection	2006	660
6	Association Between Serum 25-Hydroxyvitamin D Level and Upper Respiratory Tract Infection in the Third National Health and Nutrition Examination Survey	Ginde, AA; Mansbach, JM and Camargo, CA	Archives of internal medicine	2009	615
7	Randomized trial of vitamin D supplementation to prevent seasonal influenza A in schoolchildren	Urashima, et al.	American journal of clinical nutrition	2010	563
8	Evidence that Vitamin D Supplementation Could Reduce Risk of Influenza and COVID-19 Infections and Deaths	Grant, WB, et al.	Nutrients	2020	552
9	Unexpected actions of vitamin D: new perspectives on the regulation of innate and adaptive immunity	Adams, JS and Hewison, M	Nature clinical practice endocrinology & metabolism	2008	544
10	Vitamin D3 Induces Autophagy in Human Monocytes/ Macrophages via Cathelicidin	Yuk, JM, et al.	Cell host & microbe	2009	542

#### Keyword co-occurrence and cluster

**Figure 4:**
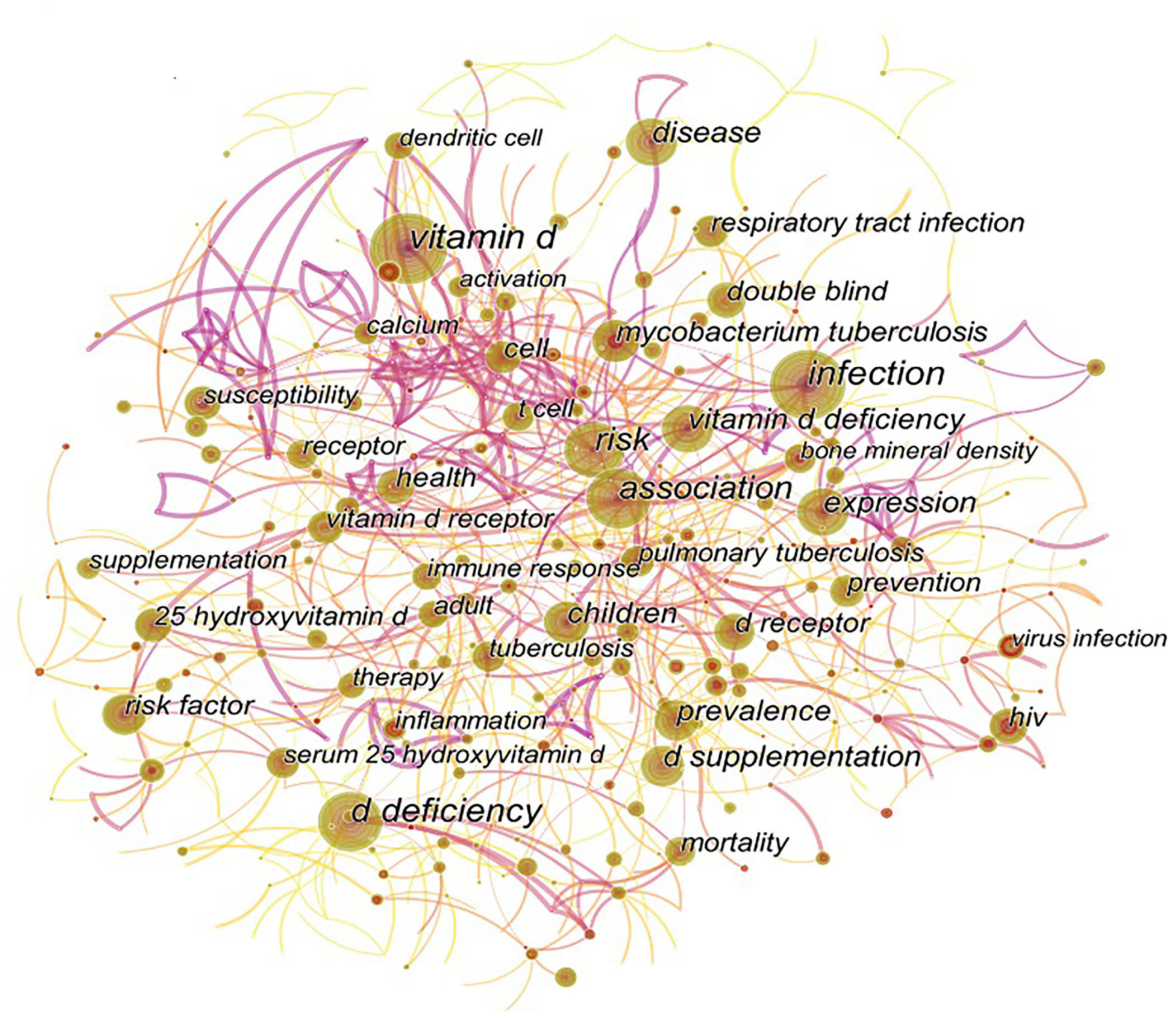
Keyword co-occurrence network (2001–2021). The nodes represent keywords, and the larger nodes indicate more research articles in that direction. The lines linking the nodes indicate the research connection between keywords, and the thicker the line, the stronger the connection. The top 10 co-occurrence keywords are “vitamin D”, “infection”, “d deficiency”, “risk”, “association”, “expression”, “disease”, “d supplementation”, “vitamin d deficiency”, and “children”.

Two or more keywords appearing in the same literature are considered as one co-occurrence. Keyword co-occurrence map is based on the frequency of keyword co-occurrence in the cited literature. The keyword co-occurrence analysis helps to identify research hotspots and predict research trends in certain fields. The keyword co-occurrence diagram is presented in Figure 4. The top 35 keywords based on the co-occurrence frequency are displayed in Table 3. As shown in Figure 4, besides vitamin D and infection, the 10 most frequent keywords from 2001 to 2021 are “d deficiency”, “risk”, “association”, “expression”, “disease”, “d supplementation”, “vitamin d deficiency”, and “children”. Centrality is a measurement of the importance of a node in network analysis. Based on the centrality ranking, the top five keywords are “cell” (centrality 0.22), “epstein barr virus” (centrality 0.2), “allele” (centrality 0.18), “susceptibility” (centrality 0.15), and “calcitriol” (centrality 0.15).

**Table 3:** Keyword co-occurrence frequency (Top 35 in count order, 2001–2021).

**Keywords**	**Count**	**Centrality**	**First appearance year**
vitamin d	594	0.01	2001
infection	502	0.01	2001
d deficiency	424	0.07	2006
risk	364	0.04	2004
association	356	0.09	2001
expression	226	0.09	2001
disease	223	0.01	2001
d supplementation	211	0.01	2009
vitamin d deficiency	192	0.07	2001
children	181	0.11	2003
prevalence	181	0.02	2007
mycobacterium tuberculosis	158	0.1	2001
risk factor	147	0.01	2002
d receptor	146	0.01	2007
double blind	140	0.03	2010
hiv	130	0	2005
cell	129	0.22	2004
health	126	0	2009
prevention	124	0	2009
respiratory tract infection	121	0.01	2010
vitamin d receptor	121	0.06	2007
25 hydroxyvitamin d	117	0.09	2006
mortality	114	0.05	2006
receptor	94	0.02	2008
adult	93	0.03	2002
pulmonary tuberculosis	91	0.08	2002
immune response	89	0.03	2007
susceptibility	88	0.15	2002
tuberculosis	87	0.03	2004
t cell	87	0.01	2007
supplementation	86	0.01	2008
serum 25 hydroxyvitamin d	85	0.06	2008
inflammation	80	0.04	2010
therapy	79	0.04	2007
calcium	78	0.1	2001

**Figure 5:**
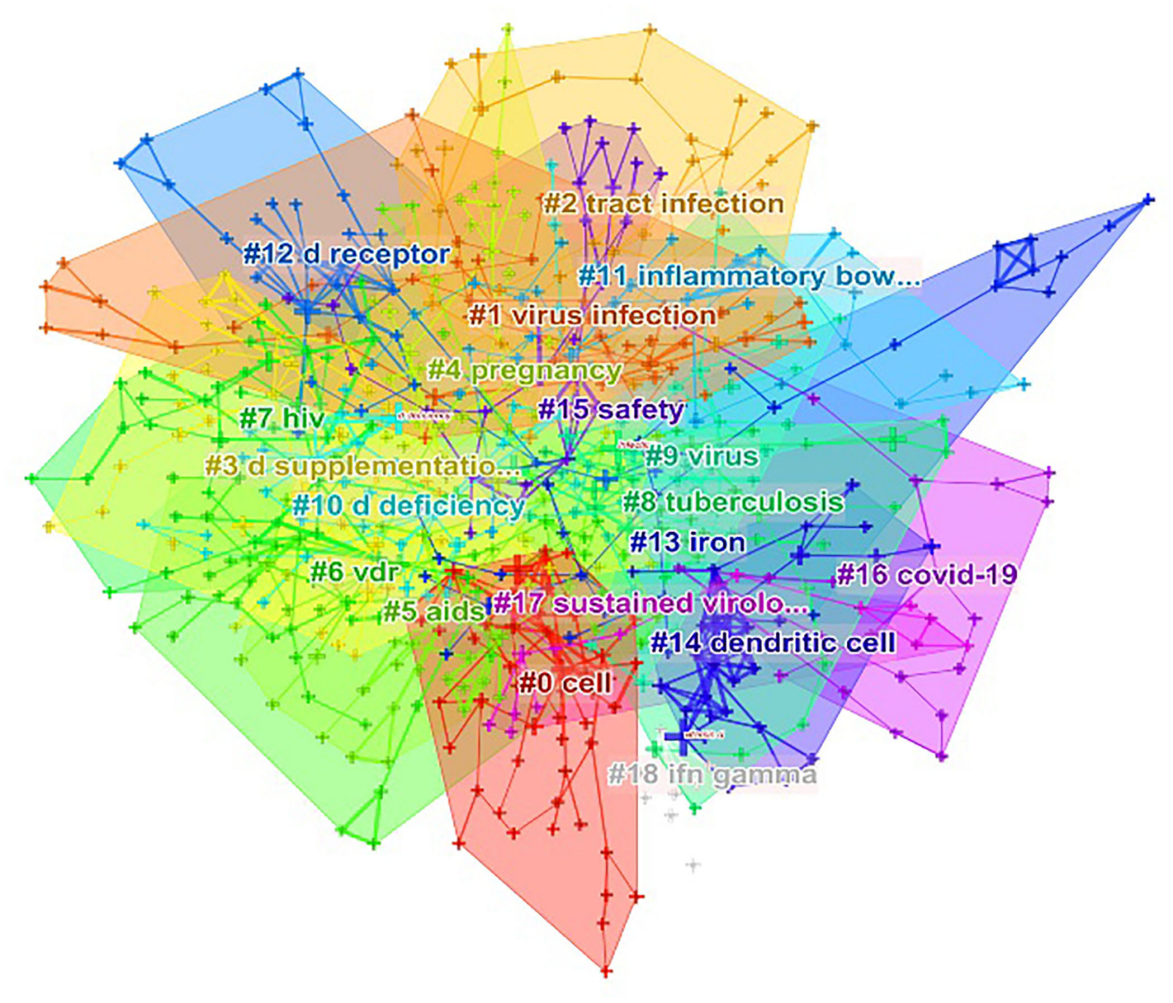
Keyword cluster analysis (2001–2021). There are 19 clusters in total and are distinguished by different colors. Cluster #0 is the largest one, followed by cluster #1, and onwards. The top three clusters are “cell”, “virus infection,” and “tract infection”.

Keyword cluster is a network of clusters formed by keywords with similar research topics to reveal the main themes. Generally, clusters are efficient and credible when silhouette > 0.7. A total of 19 distinct clusters are obtained. Within each cluster, the title word used with high frequency in the article serves as an identifier for the cluster connotation. Clusters are numbered from 0 in CiteSpace, namely, cluster #0 is the largest cluster, while cluster #1 is the next largest, and so on. According to the keyword cluster analysis, the top three clusters are “cell”, “virus infection” and “tract infection” (Figure 5 and Table 4). In cluster #0, the related keywords include “risk”, “vitamin d receptor polymorphisms” and “covid-19”. In cluster#1 “virus infection”, the most appeared keywords include “epstein barr virus”, “interferon”, “diet” and “polymorphism”. In cluster #2 “tract infection”, the major keywords mentioned are “influenza a”, “lung infection” and “rsv bronchiolitis”. “tuberculosis” ranked as cluster #8, and the newly emerged “covid-19” ranked as cluster #16.

**Table 4:** Cluster summary (19 clusters by size, 2001–2021).

**ClusterID**	Label (LLR)*	**Size**	**mean(Year)**
0	cell	49	2011
1	virus infection	44	2013
2	tract infection	43	2016
3	d supplementation	41	2011
4	pregnancy	41	2013
5	aids	40	2010
6	vdr	40	2011
7	hiv	40	2010
8	tuberculosis	37	2008
9	virus	36	2011
10	d deficiency	36	2012
11	inflammatory bowel disease	35	2013
12	d receptor	32	2006
13	iron	30	2012
14	dendritic cell	30	2005
15	safety	30	2010
16	covid-19	23	2016
17	sustained virological response	14	2013
18	ifn gamma	9	2004

* *Only the first keyword of per cluster is listed*.

#### Keyword bursts and the most recent publications

**Figure 6:**
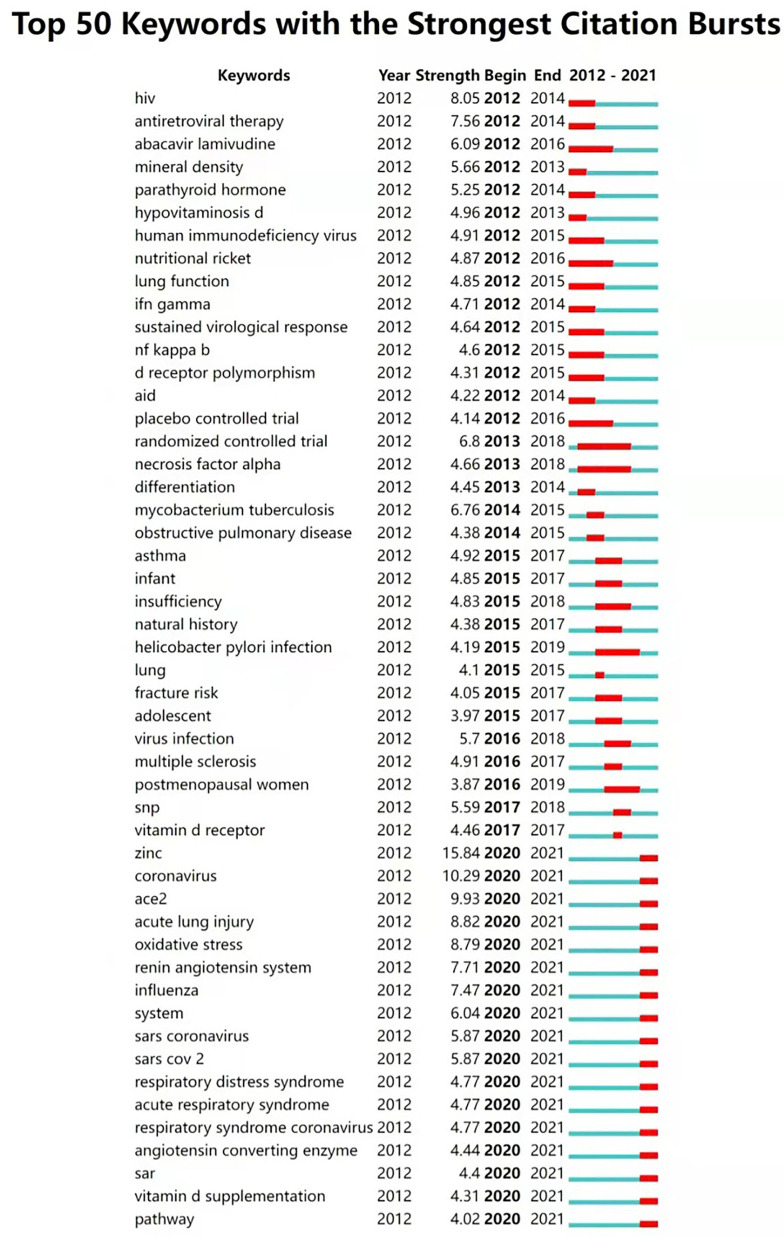
Top 50 keywords with the strongest citation burst (2012–2021). The blue line indicates the time-lapse, and the red line indicates the duration of the quote burst, which shows the progression of cutting-edge hot topics.

Keyword bursts refer to the sudden increase of keywords in a specific research area at a certain time. Combined with keyword co-occurrence and cluster analysis, it can present a more comprehensive picture of the evolution of research trends and hotspots in related fields. Figure 6 shows the list of top 50 keywords bursts during the last decade. Keywords with higher strength include “zinc” (15.84), “coronavirus” (10.29), “ace2” (9.93), “acute lung injury” (8.82), “oxidative stress” (8.79), “hiv” (8.05), “renin angiotensin system” (7.71), and “influenza” (7.47). “mycobacterium tuberculosis” also has a high strength of 6.76. Keywords with a long duration of citation burst include “randomized controlled trial” (2013-2018), “necrosis factor alpha” (2013-2018), “placebo controlled trial” (2012-2016), “abacavir lamivudine” (2012-2016). “nutritional ricket” (2012-2016), “sustained virological response” (2012-2015), “nf kappa b” (2012-2015), and “d receptor polymorphism” (2012-2015). The latest burst keywords include “coronavirus” (2020-2021), “acute lung injury” (2020-2021), “oxidative stress” (2020-2021), “renin angiotensin system” (2020-2021), and so on.

The most highly cited articles in 2021 are presented in Table 5, which are all related to COVID-19. These articles focus on the role of vitamin D in the pathogenesis of COVID-19 and the relevance to the disease, such as outcomes, severity, etc.

**Table 5:** Top cited articles in 2021 related to vitamin D and infection.

**Title**	**Corresponding Authors**	**Journal**	**Cited by**	**Impact Factor** **(2021)**
Why is COVID-19 less severe in children? A review of the proposed mechanisms underlying the age-related difference in severity of SARS-CoV-2 infections	Zimmermann, P	ARCHIVES OF DISEASE IN CHILDHOOD	121	4.973
Immune-boosting role of vitamins D, C, E, zinc, selenium and omega-3 fatty acids: could they help against COVID-19?	Stojanovska, L	MATURITAS	78	5.110
Vitamin D Status in Hospitalized Patients with SARS-CoV-2 Infection	Hernandez, Jose L.	JOURNAL OF CLINICAL ENDOCRINOLOGY & METABOLISM	66	6.134
Low vitamin D status is associated with coronavirus disease 2019 outcomes: a systematic review and meta-analysis	Li, H	INTERNATIONAL JOURNAL OF INFECTIOUS DISEASES	52	12.074
The impact of outdoor air pollution on COVID-19: a review of evidence from in vitro, animal, and human studies	Bourdrel, T	EUROPEAN RESPIRATORY REVIEW	46	9.553
The link between COVID-19 and VItamin D (VIVID): a systematic review and meta-analysis	Fuleihan, GEH	METABOLISM-CLINICAL AND EXPERIMENTAL	43	13.934
Vitamin D Deficiency Is Inversely Associated with COVID-19 Incidence and Disease Severity in Chinese	Cheng, LM	JOURNAL OF NUTRITION	43	4.687
A systematic review of COVID-19 and obstructive sleep apnoea	Miller, MA	SLEEP MEDICINE REVIEWS	39	11.401
Putative roles of vitamin D in modulating immune response and immunopathology associated with COVID-19	Sharma, A	VIRUS RESEARCH	35	6.286
Association of Vitamin D Status with SARS-CoV-2 Infection or COVID-19 Severity: a systematic review and Meta-analysis	Kazemi, A; Mohammadi, V	ADVANCES IN NUTRITION	34	11.576

## Discussion

### Research trends

According to the qualitative and quantitative investigations conducted by CiteSpace, the scientific production in the field of vitamin D and infections as well as the researchers devoted to it have been growing over the last 20 years. As presented in Figure 2, articles published since 2012 account for 78.2% of the total produced articles in 20 years. Our results are in agreement with previous studies carried out by Shi and colleagues in 2019. Their findings revealed that in recent years, especially from 2015 to 2018, the hot research topic on vitamin D-related diseases has shifted significantly from musculoskeletal-related to the non-musculoskeletal-related area, such as neuropsychological, cardiovascular disease, cancer, and infectious diseases (27). Of note, the 998 articles published during 2020 and 2021 account for nearly one-third of all published 3,046 articles in the last two decades (Figure 2), which is linked to the global outbreak of the COVID-19 epidemic in late 2019.

As illustrated in Table 1, in this field, the US, the UK, Italy, China, and India ranked the top five countries in the total number of publications. Of the top five contributing institutions, namely the League of European Research Universities, University of London, Harvard University, University of California System, and Egyptian Knowledge Bank, two belong to the United States and two in Europe. In terms of the number of individual publications, Martineau AR and Camargo CA ranked as the top two authors. Of the five authors with the highest number of published articles, two professors, Camargo CA and Sun J, work in US universities, while the rest three authors Martineau AR, Griffiths CJ, and Hewison M come from UK institutions. What is even more remarkable is that these authors have remained focused on the field for more than one decade and continue to present high-quality papers up to now. As for the top five funding agencies, three are affiliated with the United States and the rest are from Europe. Hence, our results reveal the consistency in the leading authors, institutions, and countries. Meanwhile, our study also shows the absolute leadership of the Western countries in this field, which was consistent with previous studies (28). In the early twentieth century, researchers in the Western world identified the structure of vitamin D (29) and have consistently attached importance to the study of vitamin D. Owing to the emphasis of governments, outstanding research institutions, intense academic atmosphere, and sufficient research funding, Western developed countries have made outstanding contributions in this area.

Interestingly, further analysis of the countries burst (Figure 3) shows that, in addition to the traditional academic giants, research in developing countries and Middle Eastern countries has been increasing in recent years. Compared to Western developed countries, medical facilities and healthcare framework of developing countries are still relatively lagging behind. However, vitamin D deficiency is more prevalent in low- and middle-income countries, including India and Iran (24). Meanwhile, based on studies in the Middle East, clothing style is also an essential factor in determining vitamin D levels (30). The entire skin-covered dressing style, limited outdoor activities due to the summer heat, limited vitamin D fortification, and dietary habits might explain the extremely low vitamin D concentrations in Middle East countries (31–34). In addition, although serum or plasma 25(OH)D concentrations are considered to be the most reliable biomarkers for determining vitamin D status (3), it is often difficult to monitor or obtain data of 25(OH)D in populations in low- and middle-income countries, which would hamper the effort to evaluate vitamin D status. It is expected that these studies on vitamin D will draw the attention of the relevant authorities in these countries and lead to the adoption of necessary actions.

### Research focuses

According to the results of co-occurring keywords (Figure 4) and keyword cluster (Figure 5), it is illustrated that the relationship between vitamin D and respiratory infections, especially respiratory viruses and the more recently coronaviruses, has received a lot of attention in the last two decades, suggesting that this is a hot issue for research. In addition, as shown in Figure 6, the research hotspots in this area have evolved in the last decade. 10 years ago, there was concern about the relevance of vitamin D to specific diseases (such as HIV infections, tuberculosis) and associated mechanisms (such as “sustained virological response,” “nf kappa b,” and “d receptor polymorphism”). The maturation of research in this area will lead to increasing concerns of researchers regarding the use of vitamin D for diseases' prevention or treatment. Therefore, it is not surprising that “randomized controlled trials” and “placebo controlled trials” have become a hot topic of study (Figure 6), indicating that researchers were beginning to investigate the efficacy of vitamin D applied to clinical diseases.

The most cited article (Table 2) published in *Science* by Liu and colleagues discussed the role of vitamin D in the treatment of tuberculosis (TB). It was demonstrated that Toll-like receptor (TLR) activation of human macrophages was followed by the upregulation of VDR and vitamin D-1 hydroxylase gene expression, resulting in cathelicidin induction and intracellular killing of Mycobacterium tuberculosis (35). The research further suggested that, for the first time, the increased susceptibility to tuberculosis in African Americans was associated with low serum levels of 25(OH)D, which was insufficient to sustain the induction of antimicrobial peptide cathelicidin messenger RNA. Other *in vitro* findings revealed that calcitriol mediated the response of the host to Mycobacterium tuberculosis infection by inducing reactive oxygen intermediate (36), and the antimicrobial peptide cathelicidin (37) that triggers autophagy (38).

Based on the results of many such studies, researchers have maintained a lively interest in the relationship between vitamin D and tuberculosis for the past two decades. Interestingly, these clinical trials yielded very different conclusions. A recent study in Indonesia showed that compared to the placebo group, fever and cough of TB subsided faster in the vitamin D supplementation group (39). Meanwhile, a meta-analysis that summarized the effect of vitamin D supplementation on the prognosis of patients with pulmonary TB considered it a combination therapy (40). However, another recent randomized controlled trial (RCT) conducted for 3 years showed that vitamin D supplementation did not reduce the risk of TB infection or TB disease compared to placebo among Mongolian schoolchildren who were vitamin D-deficient (41). Not only that, but also the polymorphism of VDR correlated with study results. Calcitriol regulates the immune response *via* binding to the VDR which is expressed aboard antigen-presenting cells and active lymphocytes, thereby modulating the transcription of vitamin D-responsive genes (8). Of note, human VDR carrying the *t* allele of the TaqI VDR polymorphism or the *f* allele of the FokI VDR polymorphism associates with different or even opposite performance in Mycobacterium infection (42). One of the multi-center RCTs conducted by Martineau and colleagues showed that adjunctive high-dose vitamin D_3_ (2.5mg per dose, four times in total) reduced the time to sputum culture conversion in adult TB patients with TaqI VDR polymorphism (43). Other RCTs in Mongolian adults showed that in patients carrying one or more minor variations in the gene encoding VDR, adjuvant vitamin D (one-time oral supplementation of 14,000 IU per week) speeded up the conversion of sputum cultures (44). However, in the entire study population, the supplementation of vitamin D presented no effect on the time to sputum culture conversion (44). Research on this topic continues and is controversial, which may partly explain why the keywords “d receptor” and “d receptor polymorphism” have drawn extensive attention in the last few decades (Table 3 and Figure 6).

At present, TB still ranks as the 13th cause of death and the second leading infectious disease contributor, only second to COVID-19 globally (45). In 2020, a population of 1.5 million people died from TB (45). Countries with a high TB burden accounted for the vast majority of new TB cases, with India leading the way, followed by other developing countries such as China, Indonesia, Pakistan, and South Africa. It is not surprising, therefore, that there has been a boost in the research in these countries recently (Figure 3), and we should be pleased about this. If the impact could be demonstrated in larger-scale studies, the public health implications would be clarified, as improved vitamin D status could improve innate immunity and contribute to the prevention and treatment of TB infection.

Besides TB, the relationship between vitamin D and acute respiratory infections (ARIs) has also been extensively discussed (Figures 4, 5). Several studies of RCTs reveal that vitamin D supplementation has a protective effect against influenza (46, 47). The sixth-ranked highly cited article (presented in Table 2) showed that serum 25(OH)D levels were inversely associated with acute upper respiratory tract infection (48). The association may be more significant in patients with respiratory diseases such as asthma and chronic obstructive pulmonary disease (48). Furthermore, it was suggested that children with low vitamin D status were related to a significantly higher risk of admission to the intensive care unit (ICU) and invasive mechanical ventilation (49). The underlying mechanisms of vitamin D against respiratory viral infections involve antiviral and anti-inflammatory effects, such as increased viral killing, reduced pro-inflammatory cytokine production, and protection of the integrity of tight junctions, thus keeping immune cells from invading lungs (50, 51). The second most cited literature (Table 2) also demonstrated that vitamin D deficiency was associated with an elevated risk of occurring ARIs (20). The systematic review and meta-analysis indicated that vitamin D supplementation was safe and could prevent acute respiratory infections on the whole. Amazingly, a daily or weekly regimen was more efficient than a one-time injection (20). Moreover, patients with extreme vitamin D deficiency and those who did not receive high doses of vitamin D benefited the most (20). More importantly, in 2021, researchers updated the meta-analysis of aggregated data from 48,488 participants with an age range from 0 to 95 years. The data again reported a small but significant beneficial effect of vitamin D supplementation on the association with the risk of one or more ARIs compared to placebo (52). Meanwhile, the protective effect of vitamin D was relevant to a daily dose of 400–1,000 IU for about 12 months in the 1–15.9 year age groups while was independent of different baseline 25(OH)D concentrations (52), which was in contrast to the previous findings.

Similar to the controversial relationship between TB and vitamin D, there are many different voices in the debate about the association of ARIs with vitamin D. A recent RCT showed that in young healthy Canadian children with a high 25(OH)D baseline, the high-dose vitamin D oral supplementation group (2,000 IU/day) lacked an effect on the incidence of upper respiratory tract infections compared with the regular-dose group (400 IU/day) (53). A large, double-blind, placebo-controlled D-Health Trial conducted for 5 years in Australia suggested that oral vitamin D3 (60,000 IU per month) failed to influence the incidence of upper respiratory infections (54). However, there was some benefit of taking vitamin D that patients receiving vitamin D had fewer days (0.5 days) of symptoms than those in the control group (54). Certainly, the researchers also concluded that the difference, while statistically significant, was of unclear clinical meaning. The protective effect of vitamin D remains controversial even in groups with severe vitamin D deficiency (41, 55). Since ARIs are prevalent in children younger than 5 years old (56) and lower respiratory tract infections are one of the leading causes of death in these children (57), more research is required and worth continuing in future.

### Research at the frontier and in future

As discussed in the above section, the implications of vitamin D supplementation for respiratory infections have been widely addressed, with both proponents and opponents holding their views. The emerging COVID-19 outbreak further escalates the debate. Figure 6 also confirms the trend that since 2020, research related to COVID-19 is undoubtedly the hottest topic.

Since its outbreak, COVID-19 has been of great concern worldwide. The disease is a severe lower respiratory tract viral infection which is caused by a highly infectious, severe acute respiratory syndrome coronavirus 2 (SARS-CoV-2). It is demonstrated that vitamin D possesses anti-inflammatory and antioxidant characteristics against COVID-19 infection (58). *In vitro* findings revealed that calcitriol exhibited antiviral activity toward SARS-CoV-2. Another important study showed that vitamin D attenuated lipopolysaccharide-induced acute lung injury through the renin–angiotensin system (RAS) by regulating the expression of angiotensin-converting enzyme 2 (ACE2) in rats (59). Lower 25(OH)D and 1,25(OH)_2_D levels were independently associated with upregulation of RAS activity and angiotensin 2 concentrations (60). The excessive level of RAS activation is related to a poorer prognosis of COVID-19 (61). As displayed in Table 5, the tenth-ranked article summarizes the role of vitamin D in the pathogenesis of SARS-CoV-2 and specifically highlights its modulation of the immune dysfunctional response following cytokine storm in critically ill patients (62). The multiple mechanisms by which vitamin D modulates the immune system include inhibition of SARS-CoV-2 access and replication, reduction in pro-inflammatory cytokine concentrations, increased anti-inflammatory cytokine levels, enhanced natural antimicrobial peptide production, and activation of defense cells capable of destroying SARS-CoV-2, such as macrophages (62). Since there has been a lot of research on this subject, it is understandable that keywords related to it are popular in last 2 years (Figure 6).

Interestingly, in contrast to other respiratory viral infections that tend to be prevalent in children, severe COVID-19 cases are less likely to be seen in infants and young children (63, 64). In the top-cited article of 2021 (Table 5), Zimmermann and colleagues reviewed this issue, suggesting that vitamin D was one of the important influencing factors (65). In most countries, vitamin D supplementation is routinely taken by children or infants, while vitamin D deficiency is more common in the elderly (66). According to the investigation from 2011 to 2014, the prevalence of deficient and inadequate risk of vitamin D in the United States was lowest in kids aged 1–5 years (23).

Researchers were also interested in the correlation between low vitamin D levels and the severity, incidence, and mortality of COVID-19. Of the highly cited articles in 2021 (Table 5), five out of 10 are on this issue (ranked third (67), fourth (68), sixth (69), seventh (70), and 10 (71), respectively) but there are certain differences in their conclusions. In general, the association between lower vitamin D status and COVID-19 is affirmed and it is further acknowledged that vitamin D deficiency may increase the risk of COVID-19 incidence (67–71). However, the correlation between vitamin D and the severity of COVID-19 is conflicting. The meta-analysis conducted by Kazemiand and colleagues suggested that although the results on the relationship between vitamin D deficiency and ICU admissions, pulmonary comorbidities, and hospitalizations were not consistent among studies, most of them showed a positive correlation between 25(OH)D and COVID-19 severity and mortality (71). Cheng and colleagues also indicated that vitamin D deficiency affected COVID-19 hospitalization and severity among the Chinese population (70). However, other two studies (67, 69) took the opposite view that the severity of COVID-19 and vitamin D deficiency was not relevant. It was also suggested that vitamin D supplementation may be protective against COVID-19-related ICU admissions (69), especially in frail older adults (72–74), although more solid evidence was needed as also suggested by previous bibliometric analysis (75).

In fact, the debate between these articles is also a microcosm of the research field's controversy. At present, the correlation between vitamin D deficiency and COVID-19 is still disputed, with some studies suggesting that these two are irrelevant (76–78). A systematic review and meta-analysis conducted by Ghasemian and colleagues showed that there was no significant association between vitamin D status and higher mortality rates of COVID-19 (79). In the multi-center RCT performed by Murai and colleagues, a single high oral dose of 200,000 IU cholecalciferol did not lead to a significant reduction in the hospital stay of patients with moderate-to-severe COVID-19 compared to placebo (80). Notably, participants in this study received different concomitant medications and took vitamin D for a longer period of time (mean 10.3 days) after the onset of symptoms. Therefore, it is uncertain whether the null result is related to this delayed treatment.

Actually, many variables seem to contribute to the inconsistency and discrepancy of the complicated role of vitamin D in infections. Factors, such as variations in *in vitro* and *in vivo* studies, different sample sizes and ages, different clinical trial designs, and different supplementation dose regimens, may account for the controversial results of vitamin D in the prevention and treatment of infectious diseases. It should be aware that there are fundamental differences between vitamin D RCT designs versus drug RCT designs (81, 82). One important point is that any conclusions about the health benefits of a certain dose of vitamin D supplementation must be informed by the baseline 25(OH)D concentrations in the study population and the vitamin D status achieved after treatment (81). In addition, since there are so many factors that affect vitamin D, the body concentration of vitamin D might vary, for example, seasonally. Moreover, the definition, threshold, and indicators of vitamin D deficiency also vary between countries and organizations (83). Another underestimated factor is the impact of DBP. The circulating DBP level is variable, and changes in DBP levels may affect the assessment of vitamin D status. A recent study demonstrated that while total 25(OH)D levels were significantly lower in critically ill patients, the calculated free 25(OH)D concentrations were not decreased compared to controls. Therefore, measuring only the total 25(OH)D concentration may lead to an underestimation of vitamin D status and an overestimation of the number of patients with vitamin D deficiency (84). Meanwhile, DBP polymorphisms may be associated with COVID-19 prevalence and mortality (85). Median plasma concentrations of 25(OH)D also depend on DBP polymorphisms (86). Further studies are needed to investigate the association between DBP and vitamin D status. In general, despite some developments, our knowledge in this area still lags far behind. The mechanism of vitamin D involvement in the immune system is not fully elucidated. Therefore, the deciphering of the causality of vitamin D in the development of infections remains challenging. Although we are glad to see that new data on the health benefits of vitamin D continue to emerge in this field, more hypothesis-driven studies are required in future.

Developments in this field have also led to the updating of vitamin D guidelines. In June 2020, the Scientific Advisory Committee on Nutrition (SACN) released a rapid review on vitamin D and acute respiratory tract infections and concluded that the evidence was insufficient to support vitamin D supplementation to specifically prevent ARIs in the general UK population. Interestingly, in December 2020, SACN updated the rapid review and concluded that “there may be some benefit from daily, low-dose vitamin D supplementation” in reducing risk of ARIs (87). These recommendations are also consistent with the UK government guidelines launched on 22 December 2020, granting people at high risk from COVID-19 the option to receive 4 months of daily 10 microgram (400 IU) vitamin D supplements for free (88).

It should be aware that vitamin D deficiency is relatively common in individuals of all age groups worldwide (89). Dietary patterns, mandatory supplementation strategies, age, latitude, urbanization, air pollution, sunscreen usage, lifestyle, skin pigmentation, and genetic factors are all associated with vitamin D status (13, 22, 89, 90). Interestingly, studies showed that vitamin D did not impact the immune effect of flu vaccines (91, 92). Thus, vitamin D supplements may be more convenient and acceptable as a way to prevent the flu than medications and flu vaccines because of their safety and many other benefits for healthy skeletons. Moreover, despite the tremendous efforts of frontline health workers, morbidity and mortality of COVID-19 continue to rise globally. While in an ideal world all health decisions should be grounded on overwhelming proof, times of crisis may require a slightly different set of rules as well as more prompt judgment, and certainly, solid evidence remains a necessity.

Considering the wide range of populations affected by vitamin D deficiency, the multiple diseases associated with it, and the relatively simple means of supplementation, it is still worthwhile to continue research in this area. Even a small experimental benefit will be of significant public health importance in a large-scale population. Special attention should also be paid to those who are prone to vitamin D deficiency, many of whom are associated with poorer clinical outcomes. Authorities should be aware of the issues and take initiatives to improve the health status of the population and consequently reduce the burden on health care resources and society.

### Strengths and limitations

CiteSpace is not a complete replacement for systematic retrieval and still has certain limitations to be addressed. First, we obtained the literature data through the WoSCC database, but with the constant updating of the database, there is some discrepancy between the results of this study and the actual number of literature available now. Second, this study included only articles and reviews, and the quality of the literature collected was mixed. Third, only English papers were selected for this study. The above reasons may render our analysis not so comprehensive. Besides some limitations, the literature-based visual analysis still certainly provides a basis for scholars to understand the research objects, hotspots, and trends in the field of vitamin D and infection rapidly.

## Conclusion

Although the role of vitamin D on bone health has long been widely recognized, its ability to modulate immune responses and attenuate acute infectious processes has been emphasized in the last 20 years. Our article presents the developments in the field over the past two decades, from the impact of vitamin D on pathogenesis to the effects on therapeutic outcomes in various infectious diseases. Our results indicate the significant contribution of developed Western countries in this field, as well as the increasing number of countries/regions engaged in the subject of research. Despite numerous encouraging and promising findings, there is not yet a consensus on the role of vitamin D in the prevention and control of infectious diseases. As the COVID-19 epidemic keeps escalating, the overall context of the immunomodulatory effects of vitamin D in infections deserves further investigation.

## Data Availability Statement

The original contributions presented in the study are included in the article/supplementary material, further inquiries can be directed to the corresponding author.

## Author contributions

XL had the idea for the article and collected data. XL and YD performed the search and analysis. WH prepared the draft of the manuscript. XL critically revised the work. All authors contributed to the article and approved the submitted version.

## Funding

The study was supported by the Scientific Research Project of Hunan Provincial Health Commission (202117010086) and Natural Science Foundation of Hunan Province, China (Grant No. 2021JJ40832).

## conflict of interest

The authors declare that the research was conducted in the absence of any commercial or financial relationships that could be construed as a potential conflict of interest.

## Publisher's note

All claims expressed in this article are solely those of the authors and do not necessarily represent those of their affiliated organizations, or those of the publisher, the editors and the reviewers. Any product that may be evaluated in this article, or claim that may be made by its manufacturer, is not guaranteed or endorsed by the publisher.
